# Evaluation of Replication of Variants Associated with Genetic Risk of Otitis Media

**DOI:** 10.1371/journal.pone.0104212

**Published:** 2014-08-04

**Authors:** E. Kaitlynn Allen, Ani Manichaikul, Wei-Min Chen, Stephen S. Rich, Kathleen A. Daly, Michèle M. Sale

**Affiliations:** 1 Center for Public Health Genomics, University of Virginia, Charlottesville, Virginia, United States of America; 2 Department of Biochemistry and Molecular Genetics, University of Virginia, Charlottesville, Virginia, United States of America; 3 Department of Public Health Sciences, University of Virginia, Charlottesville, Virginia, United States of America; 4 Department of Otolaryngology, University of Minnesota, Minneapolis, Minnesota, United States of America; 5 Department of Medicine, University of Virginia, Charlottesville, Virginia, United States of America; Vanderbilt University, United States of America

## Abstract

The first Genome Wide Association Study (GWAS) of otitis media (OM) found evidence of association in the Western Australian Pregnancy Cohort (Raine) study, but lacked replication in an independent OM population. The aim of this study was to investigate association at these loci in our family-based sample of chronic otitis media with effusion and recurrent otitis media (COME/ROM). Autosomal SNPs were selected from the Raine OM GWAS results. SNPs from the Raine cohort GWAS genotyped in our GWAS of COME/ROM had P-values ranging from *P* = 0.06–0.80. After removal of SNPs previously genotyped in our GWAS of COME/ROM (N = 21) and those that failed Fluidigm assay design (N = 1), 26 SNPs were successfully genotyped in 716 individuals from our COME/ROM family population. None of the SNP associations replicated in our family-based population (unadjusted *P* = 0.03–0.93). Replication in an independent sample would confirm that these represent novel OM loci, and that further investigation is warranted.

## Introduction

Inflammation of the middle ear, known as otitis media (OM), is a highly prevalent disease in the pediatric population worldwide. Children at higher risk of OM may develop recurrent or chronic otitis media (COME/ROM), a condition that may lead to multiple antibiotic treatments and at least one tympanostomy tube insertion surgery. Environmental factors are known to play a role in risk of OM; however, it is also known that there is a large genetic component to OM risk.

The first family study of COME/ROM showed a higher rate of OM than general population rates [1], indicating familial aggregation of OM and potential genetic contribution to risk. Estimated heritability of OM (the proportion of risk attributable to additive genetic factors) has been shown to vary from 0.2 to 0.73 in various populations [2]–[7]. Although the estimated heritability of OM is high, there have been few studies to discover putative candidate loci or genes. Identification of OM candidate genes may provide new understanding of the etiology of OM, increase precision for disease prediction as well as provide novel targets for prevention and treatment. This impact could reduce the amount of antibiotics prescribed and the number of pediatric surgeries.

Currently, there are few studies that have the ability to study the genetics of OM. The current studies of OM include a linkage study from the University of Pittsburgh (UPitt) [8], a GWAS from the University of Western Australia (UWA) [9], and linkage and GWAS from the University of Minnesota (UMN) [10], [11]. The first GWAS of OM was conducted in Western Australia using the Western Australian Pregnancy (Raine) cohort to determine variants associated with acute and chronic OM. The Raine cohort was a longitudinal birth cohort, and OM cases were defined if clinical exam indicated the presence of inflamed, retracted, or scarred tympanic membrane; middle ear effusion; or tympanostomy tube surgery in the first three years of life. Parental self-report of at least three episodes of acute otitis media (AOM) by three years could also determine affected status. The Raine cohort analysis found no genome-wide association, but did identify two genes (*CAPN14* and *GALNT14*) as strong candidates from the GWAS, and the *BPIFA* cluster of genes in a gene-based analysis. The most significantly associated SNPs were followed up in UWA's family study of OM (WAFSOM). A total of 20 SNPs within seven genes (*CAPN14*, *GALNT14*, *GALNT13*, *BMP5*, *NELL1*, *TGFB3*, and *BPIFA1*) were genotyped. None of these were found to be significantly associated with OM in the WAFSOM population. Though they did not find replication in this study, many of the candidates have biological plausibility, including *CAPN14*, *GALNT14*, and the *BPFIA* gene cluster, and warrant further investigation in an independent population of OM.

The second GWAS of OM was conducted at the University of Virginia using the UMN family population to determine variants associated with COME/ROM [11]. Children were recruited to this study if they had tympanostomy tube surgery for COME/ROM, and their family members were also recruited. Participants were considered affected if they had at least two pieces of positive evidence of COME/ROM from an ear examination from an ENT, a tympanometric test, medical record, and self-reported history [10]. As in the Raine cohort OM GWAS, no SNP exceeded genome-wide significance for association with COME/ROM. The most significantly associated SNP in this GWAS was rs1110060 in Kinesin family member 7 (*KIF7*). Significant replication of rs10497394 on chromosome 2 was found in the University of Pittsburgh (UPitt) family population of OM. This SNP is found in an intergenic region between *CDCA7* and *SP3*, and is thought to play a role in regulation by altering binding of a transcription factor, epigenetic mark, or lamina associated domain, though functional studies are needed to determine the causal nature of this region.

A hallmark of gene discovery is replication of results. Fortunately, there is strong collaboration between groups in the genetics of OM. Critical to replication is the coordinated clinical phenotyping of study populations, as a failure to replication could be due to inconsistent phenotypes used for recruitment and lack of power due to sample size. This ongoing collaboration in the genetics of OM has achieved replication of a SNP on chromosome 2 from UMN's GWAS in UPitt's family population of OM. With evidence of association in the Raine cohort GWAS of OM, it was logical to investigate replication of the most significant SNPs from the Raine GWAS in the UMN family population.

## Methods

### Ethics Statement

This study was conducted with Institutional Review Board approval at the University of Minnesota and the University of Virginia, and adhered to the tenets of the Declaration of Helsinki.

### UMN Family-based Population

The UMN family-based population of COME/ROM has been described in detail previously [1], [10]. Briefly, probands, children who had tympanostomy tube surgery for COME/ROM, and their family members were recruited. Affected status of family members was determined using four data sources including ear examination, tympanogram, medical records, and self-reported history. Individuals from 143 families with phenotypic data and DNA available were enrolled in genetic studies. The sample includes 44 families with 5–10 members, 55 families with four members, 36 trios, and 8 families with less than three members. Recruitment for the UMN family-based population had continued since the GWAS was conducted, so the study population increased in number for the Fluidigm genotyping project. The same recruitment strategy and affected status definition was used for the continued recruitment. Demographic information for the family-based population genotyped in this replication project is described in Table 1. After initial quality control measures, this sample includes 41 families with more than five members, 56 families with four members, 48 trios, and 13 families with less than three members.

**Table 1:** Participant characteristics for the University of Minnesota (UMN) family-based study population and the Western Australian Raine longitudinal birth cohort.

Trait	UMN Replication Study	Raine cohort
	Value	Value
Number of genotyped subjects	596	1491
Affected subjects	62.0% (370/596)	27.9% (416/1491)
Caucasian	95%	94%

Definition of OM in the Raine cohort GWAS was completed using clinical examination and parental self-report from the first three years of life. Cases were defined if they had presence of inflamed, retracted or scarred TM, MEE or tympanostomy tubes, and over half of cases were defined by parental report of ≥3 episodes of AOM by the age of 3 yrs.

### SNP Identification for Replication

We identified 46 autosomal SNPs to follow up in this replication study. The Raine cohort GWAS reported a list of their top 25 statistically significant SNPs from their GWAS, and a list of the top 25 statistically significant SNPs from a subset of participants with full covariate data. Three SNPs overlapped these two lists and one was located on the X chromosome, resulting in a total of 46 autosomal SNPs.

### Investigation of Replication: Data Mining and Genotyping

From these 46 SNPs, we searched our GWAS of COME/ROM data to determine if any associations had already tested. To investigate the remaining SNPs from the Raine GWAS results, we genotyped the SNPs in the UMN family population using the Fluidigm SNPtype assay platform (http://www.fluidigm.com/snptype-assays.html). Briefly, 100 ng/uL of each DNA sample was combined with Biotium 2× Fast Probe Master Mix, SNPtype Sample Loading Reagent (Fluidigm), and the reference dye ROX (Invitrogen Inc.). Each SNP type assay was mixed with 2× Assay Loading Reagent (Fluidigm). Both sample mixes and assay mixes were then loaded onto Fluidigm 96.96 Dynamic Genotyping Arrays, and nanofluidic circuitry loads and mixes the 96 loci with 96 samples in 9216 reaction chambers. Thermocycling was performed on Fluidigm's Stand-alone Thermal Cycler and fluorescence detection performed on the EP1 genotyping system (Fluidigm).

SNP rs2704219, which did not pass quality control measures, was re-genotyped after a pre-amplification step performed with the following thermocycling conditions: 95°C for 15 minutes, then 14 cycles of 5 seconds at 95°C and 4 minutes at 60°C. Pre-amplified DNA was then diluted 1∶100 in suspension buffer and genotyped as previously described.

### Quality Control Measures and Association Test

Quality control measures, association testing, and imputation methods used in the UMN GWAS of COME/ROM have been previously described [11]. Briefly, removal of SNPs was based upon filtering for poor genotype clusters, low minor allele frequency (MAF<0.01), and genotypes inconsistent with Hardy Weinberg proportions (*P*<10^−5^). Samples and SNPs with excessive Mendelian errors (>0.6%) and/or low genotype call rates (<95%) were removed. We used the imputation method implemented in the software package MACH using HapMap3 with the CEU reference population [12], [13]. This method uses Markov models to identify stretches of shared chromosomes between individuals, and then to infer intervening genotypes by contrasting study samples with densely typed HapMap samples.

Genotype data from these 26 SNPs was analyzed in 596 individuals using the nuclear family-based Transmission Disequilibrium Test (TDT) [14]. *P*-values reported in Tables 2–3 are reported without adjustment for multiple comparisons.

**Table 2:** Top SNPs from the Raine cohort GWAS that were genotyped in our GWAS of COME/ROM.

Chr	SNP	Position (hg18)	Nearest RefSeq Gene	Raine Risk Allele	Other Allele	Risk Allele Freq	OR (95% CI)	TDT P-value	Raine OR (95% CI)	Risk Allele Freq	Raine P-value_adj-PCA_
1	rs1175549	3.681587	*SMIM1*	A	C	0.84	0.81 (0.54–1.21)	0.30	1.56 (1.27–1.91)	0.76	2.65×10^−5^
2	rs13386850	31.2992	*CAPN14* *	C	A	0.09	1.21 (0.74–1.97)	0.45	1.86 (1.44–2.38)	0.10	1.32×10^−6^
2	rs2098787∧	31.15015	*GALNT14*	G	A	0.51	0.80 (0.58–1.10)	0.17	1.58 (1.28–1.97)	0.53	3.17×10^−5^
2	rs1862981∧	31.15103	*GALNT14*	T	G	0.49	1.22 (0.89–1.69)	0.22	1.60 (1.29–1.99)	0.54	2.20×10^−5^
2	rs2377445∧	106.0271	*C2orf40* *	C	T	0.71	1.14 (0.80–1.62)	0.47	1.57 (1.26–1.96)	0.70	5.20×10^−5^
4	rs10008015	106.2247	*TET2* *	C	T	0.09	1.63 (0.92–2.89)	0.09	1.66 (1.30–2.11)	0.11	3.89×10^−6^
4	rs6826919∧	113.0233	*C4orf32* *	A	G	0.42	1.18 (0.86–1.61)	0.30	1.59 (1.27–1.96)	0.43	4.05×10^−5^
6	rs1457955∧	77.46879	*IMPG1* *	G	T	0.85	0.85 (0.53–1.35)	0.48	1.85 (1.37–2.49)	0.87	4.98×10^−5^
6	rs4709819∧	164.3833	*QKI* *	A	G	0.45	1.12 (0.82–1.52)	0.48	1.59 (1.28–2.0)	0.40	2.67×10^−6^
7	rs10242197	90.09785	*CDK14* *	C	T	0.82	0.88 (0.59–1.32)	0.54	1.59 (1.27–1.98)	0.19	4.38×10^−5^
7	rs10488001	90.49398	*CDK14*	T	C	0.09	0.84 (0.47–1.50)	0.56	1.72 (1.33–2.24)	0.09	4.53×10^−5^
8	rs2882460∧	62.68845	*ASPH*	C	A	0.81	0.86 (0.61–1.23)	0.42	1.66 (1.30–2.12)	0.79	4.96×10^−5^
8	rs6471969∧	62.72939	*ASPH*	G	T	0.84	0.85 (0.58–1.24)	0.39	1.83 (1.42–2.37)	0.82	3.85×10^−6^
8	rs11990408∧	62.74755	*ASPH*	A	G	0.83	0.88 (0.61–1.28)	0.51	1.76 (1.27–2.27)	0.81	1.10×10^−5^
8	rs11787089∧	62.78339	*ASPH*	C	T	0.87	0.83 (0.56–1.24)	0.37	1.88 (1.43–2.47)	0.85	6.83×10^−6^
10	rs4575213	53.09533	*PRKG1*	C	A	0.62	0.96 (0.69–1.33)	0.80	1.41 (1.20–1.67)	0.60	4.65×10^−5^
13	rs1336708∧	101.763	*FGF14*	G	A	0.21	1.07 (0.71–1.62)	0.75	1.75 (1.33–2.27)	0.23	5.17×10^−5^
13	rs4512966∧	109.8801	*COL4A2*	C	T	0.53	0.74 (0.54–1.01)	0.06	1.55 (1.25–1.91)	0.50	5.01×10^−5^
17	rs4627412∧	76.13884	*RPTOR*	A	G	0.65	0.92 (0.65–1.28)	0.61	1.58 (1.27–1.96)	0.69	4.92×10^−5^
21	rs2839520∧	42.74273	*UBASH3A* *	G	A	0.39	1.37 (0.99–1.90)	0.06	1.59 (1.30–1.96)	0.46	1.63×10^−5^

We report our OR and allele frequency using the Risk Allele corresponding to the Risk Allele listed in the Raine cohort GWAS [9].

*Indicates SNP is intergenic and therefore reports the nearest gene.

∧ Indicates the SNP was a top genotyped SNP from a subset of Raine study participants with full covariate data.

**Table 3:** Top SNPs from Raine cohort GWAS that were genotyped in our family population of COME/ROM.

Chr	SNP	Position (hg19)	Nearest RefSeq Gene	Raine Risk Allele	Other Allele	Risk Allele Freq	OR (95% CI)	TDT P-value	Raine OR (95% CI)	Risk Allele Freq	Raine P-value_adj-PCA_
1	rs12728900	26.746807	*LIN28A*	A	G	0.20	0.90 (0.61–1.34)	0.63	1.47 (1.23–1.75)	0.25	2.28×10^−5^
2	rs13408922	31.444826	*CAPN14* *	A	C	0.12	1.10 (0.67–1.78)	0.71	1.86 (1.44–2.38)	0.10	1.32×10^−6^
2	rs13386745	31.445615	*CAPN14* *	G	A	0.12	1.10 (0.67–1.80)	0.71	1.84 (1.44–2.37)	0.10	1.63×10^−6^
2	rs330787	48.041377	*FBXO11*	C	T	0.63	0.99 (0.70–1.38)	0.93	1.43 (1.21–1.70)	0.64	4.64×10^−5^
3	rs17624623∧	61.620466	*PTPRG*	T	C	0.58	1.03 (0.74–1.43)	0.87	1.60 (1.29–1.98)	0.59	2.02×10^−5^
4	rs11097383	94.58384	*GRID2*	C	T	0.17	0.62 (0.40–0.97)	0.03	1.55 (1.25–1.92)	0.16	5.97×10^−5^
4	rs1859161	106.042692	*TET2* *	G	A	0.12	0.97 (0.61–1.54)	0.91	1.78 (1.37–2.32)	0.09	1.90×10^−5^
4	rs11940126	186.790909	*SORBS2*	A	G	0.05	0.65 (0.32–1.31)	0.22	2.09 (1.46–3.00)	0.05	5.70×10^−5^
7	rs10242197	90.259912	*CDK14* *	C	T	0.81	0.88 (0.59–1.32)	0.54	1.59 (1.27–1.98)	0.81	4.38×10^−5^
8	rs1496306	50.540708	*SNTG1* *	A	G	0.08	0.59 (0.34–1.02)	0.06	1.76 (1.36–2.28)	0.10	1.63×10^−5^
8	rs2132528	50.605655	*SNTG1* *	G	A	0.08	0.59 (0.34–1.02)	0.06	1.75 (1.35–2.26)	0.09	2.41×10^−5^
8	rs13438948	87.858778	*CNBD1* *	A	G	0.07	1.53 (0.83–2.82)	0.17	1.82 (1.39–2.39)	0.09	1.19×10^−5^
8	rs7846284	130.370235	*GSDMC* *	A	G	0.42	0.96 (0.71–1.31)	0.82	1.40 (1.19–1.65)	0.43	4.06×10^−5^
8	rs7846684	130.370414	*GSDMC* *	C	T	0.42	0.96 (0.71–1.31)	0.82	1.41 (1.20–1.65)	0.43	3.60×10^−5^
9	rs11790808	94.588498	*ROR2*	C	T	0.04	0.79 (0.36–1.73)	0.55	2.09 (1.50–2.91)	0.05	1.15×10^−5^
10	rs16919668∧	20.279158	*PLXDC2*	G	A	0.15	1.02 (0.68–1.55)	0.92	2.0 (1.43–2.78)	0.16	3.28×10^−5^
10	rs10884043	106.531703	*SORCS3*	A	G	0.77	1.18 (0.81–1.73)	0.38	1.53 (1.25–1.86)	0.74	2.74×10^−5^
10	rs4556466	106.558025	*SORCS3*	A	G	0.77	1.20 (0.82–1.73)	0.34	1.53 (1.26–1.87)	0.74	2.74×10^−5^
13	rs9564897	72.912338	*MZT1* *	T	G	0.34	0.92 (0.66–1.28)	0.61	1.43 (1.22–1.69)	0.34	1.98×10^−5^
15	rs6493973∧	58.292636	*ALDH1A2*	C	T	0.97	0.67 (0.24–1.87)	0.44	3.43 (1.90–6.20)	0.97	4.40×10^−5^
15	rs2218261∧	58.294022	*ALDH1A2*	G	A	0.97	0.67 (0.24–1.87)	0.44	3.43 (1.90–6.20)	0.97	4.40×10^−5^
15	rs2704219∧	58.328448	*ALDH1A2*	T	C	0.97	0.67 (0.24–1.87)	0.44	3.39 (1.90–6.05)	0.97	3.72×10^−5^
17	rs11658127∧	66.714282	*FAM20A* *	A	G	0.88	1.32 (0.83–2.11)	0.24	1.93 (1.42–2.61)	0.87	2.29×10^−5^
17	rs11658297∧	66.728983	*FAM20A* *	G	A	0.88	1.39 (0.87–2.20)	0.16	1.94 (1.43–2.63)	0.87	1.89×10^−5^
17	rs9911978∧	78.524407	*RPTOR*	A	G	0.68	1.02 (0.72–1.43)	0.93	1.58 (1.27–1.96)	0.69	4.92×10^−5^
20	rs17396317	31.790377	*BPIFA4P* **	A	G	0.12	1.44 (0.84–2.44)	0.18	1.62 (1.28–2.05)	0.12	5.15×10^−5^

We report our OR and allele frequency using the Risk Allele corresponding to the Risk Allele listed in the Raine cohort GWAS [9].

*Indicates SNP is intergenic and therefore reports the nearest gene.

**Indicates SNP is coding.

∧ Indicates the SNP was a top genotyped SNP from a subset of Raine study participants with full covariate data.

Power was calculated using the TDT Power Calculator [15], varying SNP MAF and genetic relative risk (GRR). We assume the number of family members is 164 and Bonferonni correction for 26 markers corresponding to nominal error rate of α* = 0.05/26 = 0.0019 in all power calculations.

## Results and Discussion

Demographic data on the UMN family-based population genotyped in this replication project and the Raine cohort are shown in **Table 1**. Initially, we searched our GWAS data for any SNPs that were already genotyped. We found no significant associations from these 21 SNPs (**Table 2**) with P-values ranging from *P* = 0.06 (rs4512966 in *COL4A2* and rs2839520, intergenic near *UBASH3A*) to *P* = 0.80 (rs4575213 in *PRKG1*). To investigate the remaining SNPs from the Raine GWAS results, we genotyped the SNPs (N = 27) in the UMN family population using the Fluidigm SNPtype assay platform, including one SNP (rs10242197) which had been genotyped in our GWAS for quality control purposes. One SNP (rs10776851 in *CAMSAP1*) did not pass Fluidigm assay design and one SNP (rs2704219 in *ALDH1A2*), did not pass quality control measures; however, rs2704219 was successfully re-genotyped using a different Fluidigm protocol to pre-amplify samples before genotyping. A total of 26 SNPs were genotyped in 596 subjects and available for analyses.

Data was analyzed using the family-based association test TDT [14]. No significant associations with any SNP with OM status were found after Bonferroni correction (significance threshold at *P* = 0.05/46 = 0.001) (**Table 3**), with nominal unadjusted P-values ranging from *P* = 0.03 (rs11097383 in *GRID2*) to P = 0.93 (rs330787 in *FBXO11* and rs9911978 in *RPTOR*). Comparing the OR observed in our GWAS versus the Raine cohort GWAS, we observed 19/46 SNPs (41.3%) with the same direction of effect. The failure to replicate the Raine cohort OM associations may be due lack of power to detect association with SNPs of modest effect, differences in phenotypic criteria and/or differences in study design. Using our family-based population, we have approximately 80% power to detect associations with SNPs with effect OR≥2 when MAF ranges from 0.2 to 0.5 (**Table 4**). Also, inclusion criteria for case classification in the Raine cohort represent a less severe end of the OM spectrum than those used in the UMN family population. Lastly, differences in study designs, i.e. family-based vs. case-control, affect detection power, and may also reflect differences in underlying genetic architecture. Notably, associations with OM found using the nested case-control design of the Raine cohort were unable to be replicated in another family-based study, WAFSOM [9].

**Table 4:** Power was computed using TDT Power Calculator [15] varying SNP minor allele frequency (MAF) and genetic relative risk (GRR).

MAF	GRR	Power
0.20	1.6	0.284
	2.0	0.773
	2.4	0.971
0.35	1.6	0.426
	2.0	0.894
	2.4	0.993
0.50	1.6	0.439
	2.0	0.890
	2.4	0.991

Due to the complexity of this disease, it is important that study sites use comparable definitions of affected status so that replication studies and meta-analyses can be successfully conducted. Though this is a significant task to achieve, the International Consortium of the Genetics of Otitis Media (OTIGEN) is striving to standardize inclusion criteria for OM studies to better understand the genetic components increasing risk for OM. Additionally, for these population studies to be more informative, recruitment of diverse populations is necessary. The majority of OM population studies thus far have recruited participants with European ancestry, but diverse populations will enable researchers to narrow regions of linkage disequilibrium (LD) containing the underlying causal variants. Another issue which causes these studies to be more difficult is that OM-free controls are very difficult to find since most children will have at least one episode of OM in the first three years of life. Trio and family studies could provide a solution to the problem of OM control availability and misclassification.

In this study, we attempted to replicate associations from the Raine cohort GWAS of OM in our family population of COME/ROM recruited at the University of Minnesota. Though we did not find any of these SNPs to be significant in our population, this study illustrates the need for larger, diverse populations recruited with standardized inclusion criteria for cases.
